# Perspectives of people with diabetes on AI-integrated wearable devices: perceived benefits, barriers, and opportunities for self-management

**DOI:** 10.3389/fmed.2025.1563003

**Published:** 2025-04-23

**Authors:** Haitham Alzghaibi

**Affiliations:** Department of Health Informatics, College of Applied Medical Sciences, Qassim University, Buraydah, Saudi Arabia

**Keywords:** artificial intelligence, wearable devices, diabetes self-management, digital health, patient perceptions, health technology adoption, cross-sectional study

## Abstract

**Abstract:**

Wearable devices that incorporate artificial intelligence (AI) have become effective instruments for managing diabetes through real-time monitoring, improved adherence, and increased person with diabetes engagement. Person with diabetes perceptions, adoption barriers, and preferences critically impact the effectiveness and widespread utilisation of these technologies.

**Aim:**

The aim of study was to investigate the perceptions of people with diabetes regarding wearable devices, emphasising their perceived advantages, challenges, and potential role in facilitating diabetes self-management.

**Methods:**

A cross-sectional study involving 418 people with diabetes was conducted, with participants recruited via online platforms and people with diabetes groups. Data were gathered through a structured questionnaire that included Likert-scale items, multiple-choice questions, and open-ended responses. Descriptive statistics were employed to analyse quantitative data, whereas qualitative responses underwent thematic analysis to discern key trends.

**Results:**

Participants demonstrated significant awareness of the primary functions of wearable devices, with 83.9% acknowledging their utility in monitoring glucose levels and physical activity. The primary advantages comprised increased adherence to medication regimens (81.9%) and heightened confidence in diabetes management (82.1%). Significant barriers were identified, including data privacy concerns (79.7%), cost issues (77.0%), and usability challenges (75.1%). Thematic analysis of open-ended responses indicated a demand for features including actionable feedback, integration with healthcare providers, and enhanced usability. Despite these challenges, 81.9% of participants indicated a willingness to adopt AI-integrated wearable devices if recommended by healthcare providers.

**Conclusion:**

The findings indicate that people with diabetes regard wearable devices as effective instruments for managing their condition, especially in terms of real-time monitoring and adherence support. Concerns regarding privacy, cost, and device usability must be addressed to enhance adoption rates. These insights can inform the development of patient-centered wearable devices and guide healthcare strategies for the effective integration of these technologies into diabetes care.

## Introduction

Chronic diseases, including diabetes, present considerable challenges for people with diabetes and healthcare systems, necessitating continuous management and compliance with treatment protocols (1). Wearable devices have recently become important instruments in the management of chronic diseases, with the potential to enhance health outcomes and overall quality of life (1, 2). These devices, frequently incorporating artificial intelligence (AI), facilitate real-time monitoring, personalised care, and proactive health management, resulting in enhanced person with diabetes engagement and empowerment (1, 3–5).

Person with chronic conditions generally view wearable devices as advantageous. Research indicates their capacity for continuous monitoring and real-time feedback, thereby improving timely interventions and promoting a sense of control in health management (1, 3). Wearables facilitate seamless access to health data, thereby promoting active people with diabetes participation in care and enabling informed decisions regarding health behaviours (1, 6). Smartwatches connected to continuous glucose monitoring (CGM) systems enable individuals to monitor their glucose levels discreetly, thereby enhancing adherence to prescribed protocols and alleviating the challenges associated with disease management (6).

People with diabetes recognise the distinct advantages provided by wearable devices, including real-time health monitoring, seamless integration of data with healthcare systems, and facilitation of lifestyle modifications (7). These features allow for prompt interventions, support informed decision-making, and promote beneficial behavioural changes (8, 9). Motivational tools, including goal tracking and achievement notifications, enhance adherence to healthy habits, especially in the context of diabetes management (10–12). Nonetheless, despite these benefits, various challenges need to be addressed to facilitate widespread adoption and sustained utilisation of wearable technologies (13, 14).

Data privacy and security concerns constitute a major obstacle to people with diabetes acceptance of wearable devices (14–16). Numerous individuals express concern regarding the safeguarding of their health information and the possible misuse of such data (17). Usability challenges, especially among older adults or individuals with limited technological skills, underscore the necessity for intuitive designs and comprehensive user education (18). The accuracy and reliability of data produced by wearables are essential for their effectiveness, with people with diabetes s highlighting the necessity of validated and robust technologies (19, 20).

Wearable devices are widely regarded as user-friendly and convenient, despite existing concerns (21, 22). Smart socks and their corresponding mobile applications are frequently characterised by their comfort and user-friendliness, offering a more passive method of disease management that is particularly advantageous for older adults (23). Wearable devices enable people with diabetes to engage actively in their care, facilitating adherence to treatment recommendations and enhancing health outcomes. Research demonstrates that these technologies can elevate physical activity levels and improve the management of type 2 diabetes, resulting in significant health benefits (2, 3, 7).

People with diabetes express significant satisfaction with wearable devices; however, factors such as alert intrusiveness, limited design options, cost, and discomfort may impede their widespread adoption (24). Addressing these challenges and ensuring the privacy, affordability, and usability of wearable technologies are critical for optimising their role in chronic disease management. Wearable technologies can transform healthcare delivery and enhance the quality of life for individuals with chronic conditions by utilising the strengths of these devices and addressing patient concerns.

Wearable health technologies leveraging artificial intelligence (AI) are transforming diabetes management by enabling real-time glucose monitoring, predictive analytics, and personalised health interventions (25, 26). This study specifically examines AI-integrated wearable devices such as CGMs, AI-enhanced smartwatches, smart insulin pens, and AI-powered mobile health applications designed to support diabetes self-care. These technologies employ AI-driven algorithms to analyse patient data, optimise insulin dosing, and provide personalised feedback, ultimately improving disease management and patient adherence (26, 27).

### Aim of the study

To evaluate the perceptions, potential benefits, and challenges associated with the adoption of wearable devices integrated with artificial intelligence for managing diabetes among people with diabetes.

#### Study objectives

- Investigate people with diabetes’ awareness and familiarity with wearable devices for diabetes management.
- Explore people with diabetes’ perceptions of the potential advantages of wearable devices.
- Examine people with diabetes’ reported barriers to adoption.
- Provide insights into how wearable devices can be optimized to better meet the needs of people with diabetes with diabetes.

##### What this study adds

- This study offers an in-depth analysis of people with diabetes’ perceptions, emphasising the advantages and difficulties associated with the use of AI-integrated wearable devices for diabetes management.
- It identifies significant barriers including cost, data privacy, usability, and device accuracy that must be addressed for wider adoption.
- The findings highlight the significance of wearable devices for real-time health monitoring, enhanced adherence to treatment plans, and improved communication between people with diabetes and healthcare providers.
- The study emphasises patient-cantered insights, providing actionable recommendations for device developers, healthcare providers, and policymakers aimed at improving the functionality, usability, and affordability of wearable devices. These enhancements may facilitate personalised and proactive diabetes management while addressing patient concerns.
- The study highlights the significance of patient education and digital literacy in enhancing confidence and engagement with wearable technology, thereby facilitating its integration into chronic disease management.

## Methods

In this study, the term *AI-integrated wearable devices* specifically refers to four categories of smart health technologies that are commonly utilised in the management of diabetes. These include: (1) Continuous Glucose Monitors (CGMs), which continuously track blood glucose levels through interstitial fluid analysis and provide real-time alerts and feedback; (2) Smart Insulin Pens, which are digital insulin delivery tools capable of recording injection data and offering dosage optimisation support based on individual glucose trends; (3) AI-enabled Smartwatches, which are multifunctional wearable devices that monitor various health parameters such as glucose levels, physical activity, sleep patterns, and heart rate; and (4) AI-powered Mobile Health (mHealth) Applications, which are smartphone-based platforms integrated with wearable technologies, offering AI-driven insights, predictive alerts, and medication reminders. These tools were selected for inclusion in the study based on their clinical relevance, accessibility to people with diabetes, and their integration of artificial intelligence features that support personalised, real-time diabetes self-management.

### Research design

This study employed a cross-sectional research design to investigate people with diabetes’ perceptions, trust, and awareness of AI-integrated wearable devices in diabetes management. These devices incorporate real-time monitoring, predictive analytics, and AI-driven insights to support glucose regulation, insulin dosing, medication adherence, and overall disease management. The study specifically examined the following AI-integrated wearable technologies:

1. Continuous Glucose Monitors (CGMs): These devices continuously monitor glucose levels through real-time interstitial fluid analysis. They provide alerts for hyperglycaemia and hypoglycaemia events and integrate with digital health platforms to facilitate diabetes self-management.
2. Smart Insulin Pens: AI-enhanced insulin pens that record injection data, calculate optimal dosage recommendations, and provide feedback based on glucose trends and historical data, thus improving treatment adherence.
3. AI-Enabled Smartwatches: Wearable devices that incorporate glucose-tracking capabilities alongside heart rate monitoring, physical activity tracking, and sleep monitoring. These devices leverage AI-driven insights to provide personalised diabetes care.
4. AI-Powered Mobile Health (mHealth) Applications: Digital platforms utilising machine learning algorithms to analyse glucose trends, detect fluctuations, predict health risks, and provide medication reminders, ultimately enhancing adherence and self-management.

These technologies were selected based on their established role in diabetes care and their capacity to enhance self-management through AI-powered functionalities, such as real-time health tracking, medication reminders, integration with healthcare systems, and personalised feedback.

### Population and sampling strategy

The study targeted adults diagnosed with type 1 and type 2 diabetes, with varying levels of familiarity and experience with wearable health technologies. A total of 418 participants were recruited through web-based outreach, clinic announcements, and patient advocacy groups. A convenience sampling strategy facilitated rapid participant selection, while a stratified sampling approach ensured proportional representation based on diabetes type, frequency of wearable device usage, and prior experience with AI-powered health tools.

To ensure the reliability and relevance of responses, specific inclusion and exclusion criteria were applied. Participants were eligible if they met the following criteria:

- Aged 18 years or older.
- Had a confirmed diagnosis of type 1 or type 2 diabetes.
- Possessed at least a basic understanding of wearable health technologies.

Individuals diagnosed with other chronic diseases were excluded to maintain a clear focus on diabetes management. Furthermore, individuals with severe cognitive impairments that could hinder their ability to provide informed consent or accurately complete the questionnaire were also excluded. Participants unwilling to provide informed consent were not included in the study.

The recruitment period spanned 2 months, beginning in February 2024, with reminder messages sent during the third and sixth weeks of data collection to maximise participation. The recruitment strategy aimed to capture a diverse sample reflecting different levels of experience with wearable health technologies. By leveraging online platforms and clinic-based announcements, the study successfully engaged participants from a range of demographic backgrounds, ensuring a comprehensive assessment of AI-driven wearable health technologies.

### Data collection instrument

A structured digital questionnaire was the primary data collection instrument, designed to evaluate participants’ experiences, knowledge, and perceptions of AI-integrated wearable devices. The survey explicitly referenced and assessed four main device categories: CGMs, smart insulin pens, AI-enhanced smartwatches, and AI-powered mobile applications. The questionnaire consisted of five sections, each focusing on a specific aspect of AI-integrated wearables in diabetes management:

1. Assurance Letter: This section provided an overview of the nature of the study, outlining its objectives and ethical considerations. It assured participants that their participation was entirely voluntary and emphasised the value of their contributions. It also specified that completing the questionnaire would take approximately 15–20 min. Furthermore, this section detailed how participant data would be treated with strict confidentiality, ensuring anonymity and protection in accordance with ethical research standards.
2. Demographic Information: Collected data on age, gender, educational level, employment status, diabetes type, and familiarity with wearable technologies.
3. Perceptions of AI-Integrated Wearable Devices: Included 18 Likert-scale items (ranging from 1 = Strongly Disagree to 5 = Strongly Agree), assessing participants’ beliefs regarding personalised care, data accuracy, patient engagement, human interaction, technical reliability, and the overall usefulness of wearable devices in diabetes management.
4. Awareness and Knowledge Assessment: Utilised multiple-choice questions to evaluate participants’ awareness of AI functionalities, device-specific knowledge, and understanding of various wearable health technologies.
5. Opinions on AI-Driven Healthcare Tools: Explored perceptions of AI-driven healthcare solutions, such as virtual health assistants and AI-powered chatbots, with a focus on their potential to reduce clinician workload, facilitate remote consultations, and enhance real-time diabetes management.

Additionally, an open-ended question was included to elicit qualitative insights, allowing participants to describe how wearable devices could enhance diabetes self-management. Responses were expected to highlight key functionalities such as medication reminders, glucose trend tracking, automated alerts, and integration with healthcare providers.

### Validity and reliability of the data collection instrument

The questionnaire was developed following an extensive review of relevant literature and underwent a pilot study involving eight participants with diabetes to ensure clarity and relevance. Insights from the pilot study prompted minor modifications, including refinements in question wording and adjustments to enhance clarity and contextual relevance. The internal reliability of the Likert-scale items was assessed using Cronbach’s alpha, confirming strong internal consistency and ensuring the robustness of the measurement instrument.

### Data collection process

Data collection was conducted over 2 months, commencing in February 2024, via a secure online survey platform (Google Forms) to ensure confidentiality and ease of access. The survey link was disseminated through email, social media, and clinic-based patient networks to maximise participant reach. Reminder messages were sent in the third and sixth weeks of data collection to improve response rates.

To maintain a focused research scope, the study enforced strict inclusion and exclusion criteria. Eligible participants were required to be 18 years or older, have a confirmed diagnosis of diabetes (type 1 or type 2), and possess at least a basic understanding of wearable health technologies. Individuals diagnosed with other chronic diseases or with severe cognitive impairments were excluded to ensure the reliability of responses.

The structured data collection process was designed to ensure the reliability and validity of participant responses, enabling a comprehensive examination of the benefits, limitations, and adoption barriers associated with AI-integrated wearable devices in diabetes care.

### Data analysis

Quantitative data were analysed using SPSS (version 29) and R (version 4.3.0). Descriptive statistics, including frequencies, percentages, and mean scores, were calculated to summarise participants’ levels of awareness, trust, and perceived usefulness of AI-integrated wearable devices. The internal reliability of the Likert-scale items was assessed using Cronbach’s alpha, confirming acceptable measurement consistency. While the primary focus was on descriptive trends, limited inferential analyses such as correlation tests and basic regression modelling were employed to explore potential relationships between participant characteristics and their perceptions. However, more advanced statistical methods, such as chi-square tests or logistic regression, were not conducted. As a result, the study does not establish statistically significant associations or predictive factors, which constrains the ability to draw definitive conclusions about demographic predictors of acceptance.

The analysis specifically focused on four categories of AI-powered tools identified in the survey: Continuous Glucose Monitors (CGMs), Smart Insulin Pens, AI-enabled Smartwatches, and AI-powered Mobile Health Applications. These tools were referenced in both Likert-scale items and multiple-choice questions to assess awareness, perceptions, and usage patterns.

For qualitative data, thematic analysis was performed on open-ended responses to identify key patterns and emerging insights regarding participants’ perceptions of AI-integrated wearable technologies. Recurring themes were systematically coded to provide a deeper understanding of participant experiences and complement the quantitative findings. The integration of both quantitative and qualitative methodologies enhanced the interpretative depth of the study, facilitating a nuanced analysis of patient engagement with AI-driven wearable health tools.

## Results

The demographic characteristics of the study participants are presented in Table 1, including age, type of diabetes, education level, employment type, and gender. The largest group of participants was aged 56–65 years, comprising 34.4%, followed by the 46–55 years age group at 20.1%. Participants aged 26–35 years constituted 16.3%, while those aged 36–45 years accounted for 15.0% of the sample. Younger participants aged 18–25 represented 5.5%, and those aged 66–75 years made up 13.4%. This distribution reflects a predominance of middle-aged to older individuals in the study, aligning with the population most likely to utilize wearable devices for diabetes management.

**Table 1:** Demographic characteristics of study participants (*N* = 418).

Variable	Category	Count
Age	18–25	23 (5.5%)
	26–35	68 (16.3%)
	36–45	63 (15.0%)
	46–55	84 (20.1%)
	56–65	144 (34.4%)
	66–75	56 (13.4%)
Diabetes type	Diabetes type 1	248 (59.33%)
	Diabetes type 2	170 (40.67%)
Education level	Graduate	294 (70.33%)
	Postgraduate	68 (16.27%)
	Secondary school	52 (12.44%)
	Elementary school	3 (0.72%)
	Primary school	1 (0.24%)
Employment type	Government employee	223 (53.35%)
	Unemployed	89 (21.29%)
	Retired	63 (15.07%)
	Private sector employee	43 (10.29%)
Gender	Male	265 (63.40%)
	Female	153 (36.60%)

Type 1 diabetes was more prevalent among participants, accounting for 59.33%, in contrast to Type 2 diabetes, which comprised 40.67%. The majority of participants held graduate degrees (70.33%), while smaller percentages possessed postgraduate qualifications (16.27%) or had completed secondary education (12.44%). A minimal number of participants possessed elementary (0.72%) or primary school education (0.24%), suggesting a predominantly educated sample.

A significant portion of the participants were employed, with government employees constituting 53.35% of the sample. Unemployed individuals comprised 21.29%, whereas retired participants and those employed in the private sector constituted 15.07 and 10.29%, respectively. The sample comprised 63.40% males and 36.60% females, demonstrating a greater participation rate among males.

**Figure 1:**
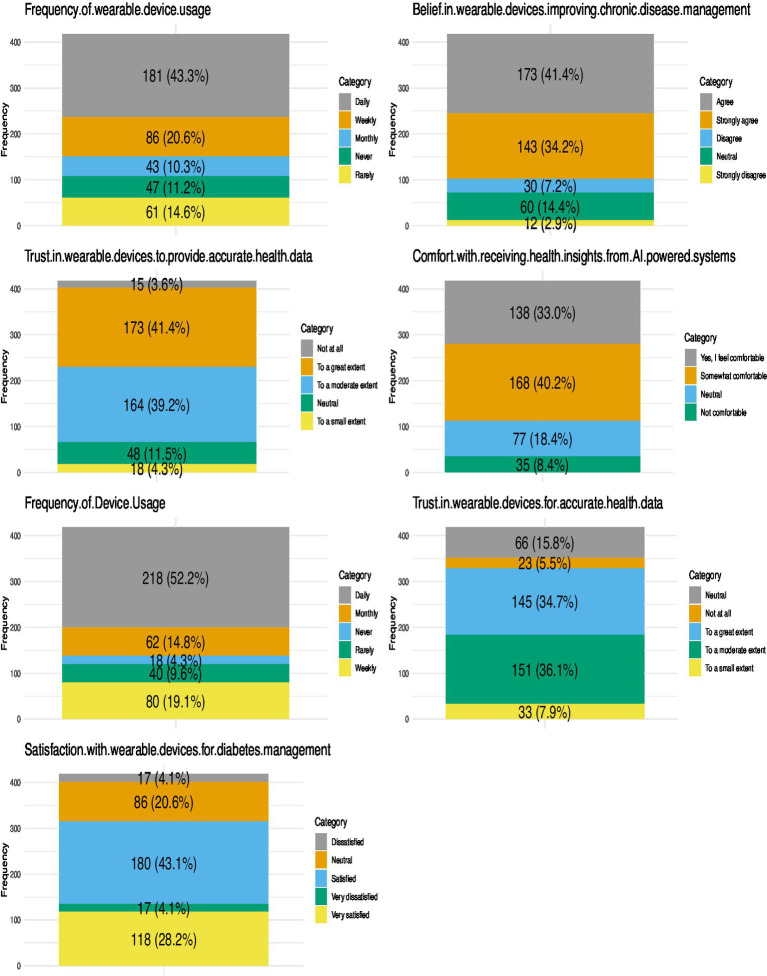
Frequency of use, perceived trustworthiness, comfort with AI-powered systems, and satisfaction levels regarding wearable devices among people with diabetes.

Figure 1 highlighted the usage, awareness, and perceptions of wearable devices among people with diabetes in seven critical domains.

### Frequency of wearable device usage

Of the participants, 43.3% indicated daily use of wearable devices, whereas 20.6% reported weekly usage. A minority reported monthly usage (10.3%) or infrequent use of wearable devices (11.6%), while 14.4% indicated they had never utilised these devices. The findings indicate that the use of wearable devices is common, with a significant portion of individuals incorporating these devices into their daily health management practices.

### Belief in wearable devices improving chronic disease management

A majority of participants concurred that wearable devices enhance the management of chronic diseases. A total of 78.3% either agreed or strongly agreed with this statement, whereas a minor proportion disagreed (7.4%). This underscores a significant conviction among people with diabetes regarding the capacity of wearable devices to improve chronic disease management.

### Trust in wearable devices to provide accurate health data

Participants exhibited a moderate to high level of trust in the accuracy of wearable devices. A total of 80.5% indicated trust to a significant degree, with 41.4% expressing great trust and 39.2% moderate trust. Nonetheless, 11.5% expressed trust to a limited degree, while 3.6% indicated a complete lack of trust. The findings indicate that although trust in the accuracy of wearable devices is predominantly high, a minority of people with diabetes exhibits scepticism.

### Comfort with receiving health insights from AI-powered systems

People with diabetes exhibited a high level of comfort in receiving health insights from AI-powered wearable systems. A majority of participants indicated a high level of comfort with this feature, with 33% reporting being very comfortable and 40.2% comfortable. Nevertheless, 18.4% reported being somewhat comfortable, while 8.4% indicated discomfort. This suggests widespread acceptance of AI-driven health insights while highlighting the necessity of addressing concerns among users who are less comfortable.

### Device usage frequency

In terms of device-specific usage, 52.2% of participants indicated daily use, while 14.8% reported weekly use and 9.1% monthly use. A significant 23.9% reported infrequent or no usage of wearable devices, indicating a necessity for enhanced accessibility or awareness among less frequent users.

### Satisfaction with wearable devices for diabetes management

Participants reported a high level of satisfaction with wearable devices utilised for diabetes management. Of the respondents, 43.1% reported satisfaction, 28.2% indicated high satisfaction, 20.6% remained neutral, and 8.1% expressed dissatisfaction. Most participants perceive wearable devices as advantageous for diabetes management; however, there remains an opportunity to address the concerns of less satisfied users.

### Trust in wearable devices for accurate health data

Trust in the accuracy of health data provided by wearable devices was affirmed, with 41.4% of participants reporting high trust and 39.2% reporting moderate trust. A minority (15.1%) indicated trust to a limited degree, highlighting the necessity to enhance perceptions of device reliability.

**Figure 2:**
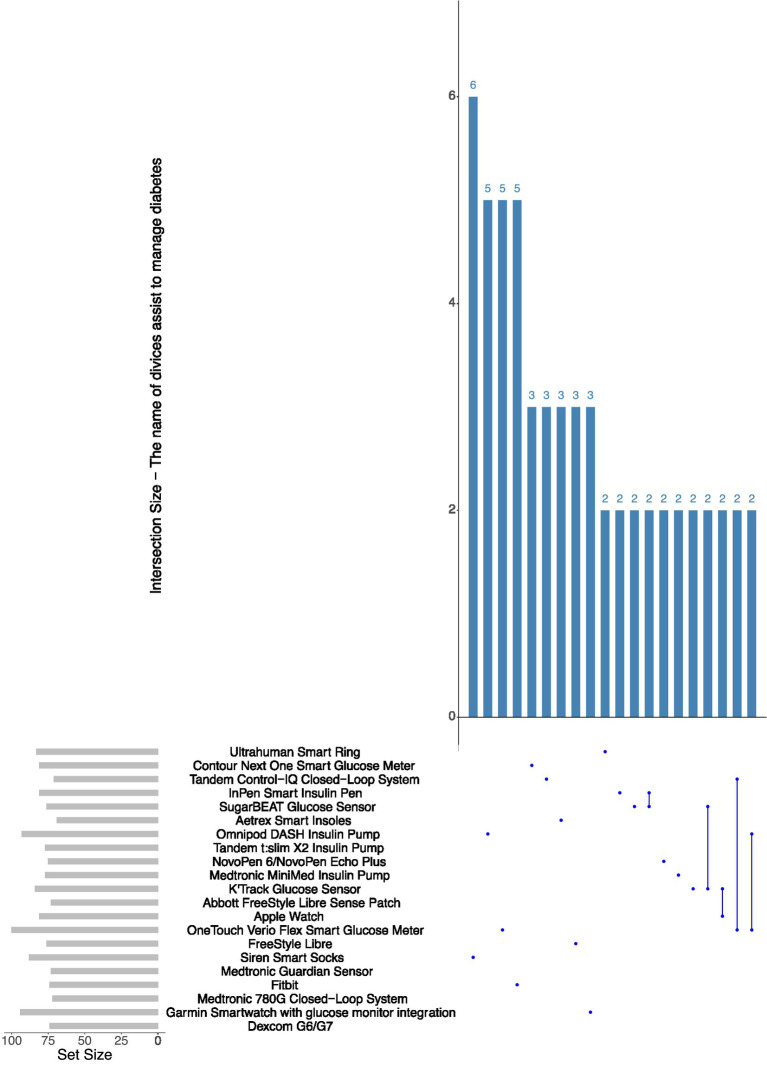
Frequency of mentioned device models used for diabetes management.

The upset plot illustrates people with diabetes’ awareness of wearable devices commonly utilised in diabetes management, offering insights into the recognition of specific devices and their combinations. CGM and smartwatches were the devices most commonly acknowledged, with 180 and 170 participants, respectively, indicating awareness. The prominence of these devices can be attributed to their extensive use in diabetes management and their integration with advanced health-tracking technologies (see Figure 2).

The plot reveals significant intersections among devices. One hundred twenty participants indicated awareness of both continuous glucose monitors and smartwatches, highlighting a significant overlap in familiarity with these devices. Ninety participants demonstrated awareness of continuous glucose monitors, smartwatches, and fitness trackers, indicating the growing adoption of various wearable technologies among people with diabetes. Intersections involving unconventional devices, such as smart shoes or smart clothing, exhibited significantly smaller sizes, with fewer than 20 participants acknowledging combinations that included these technologies. This suggests a lack of awareness and adoption of emerging wearable devices within the diabetic population.

**Figure 3:**
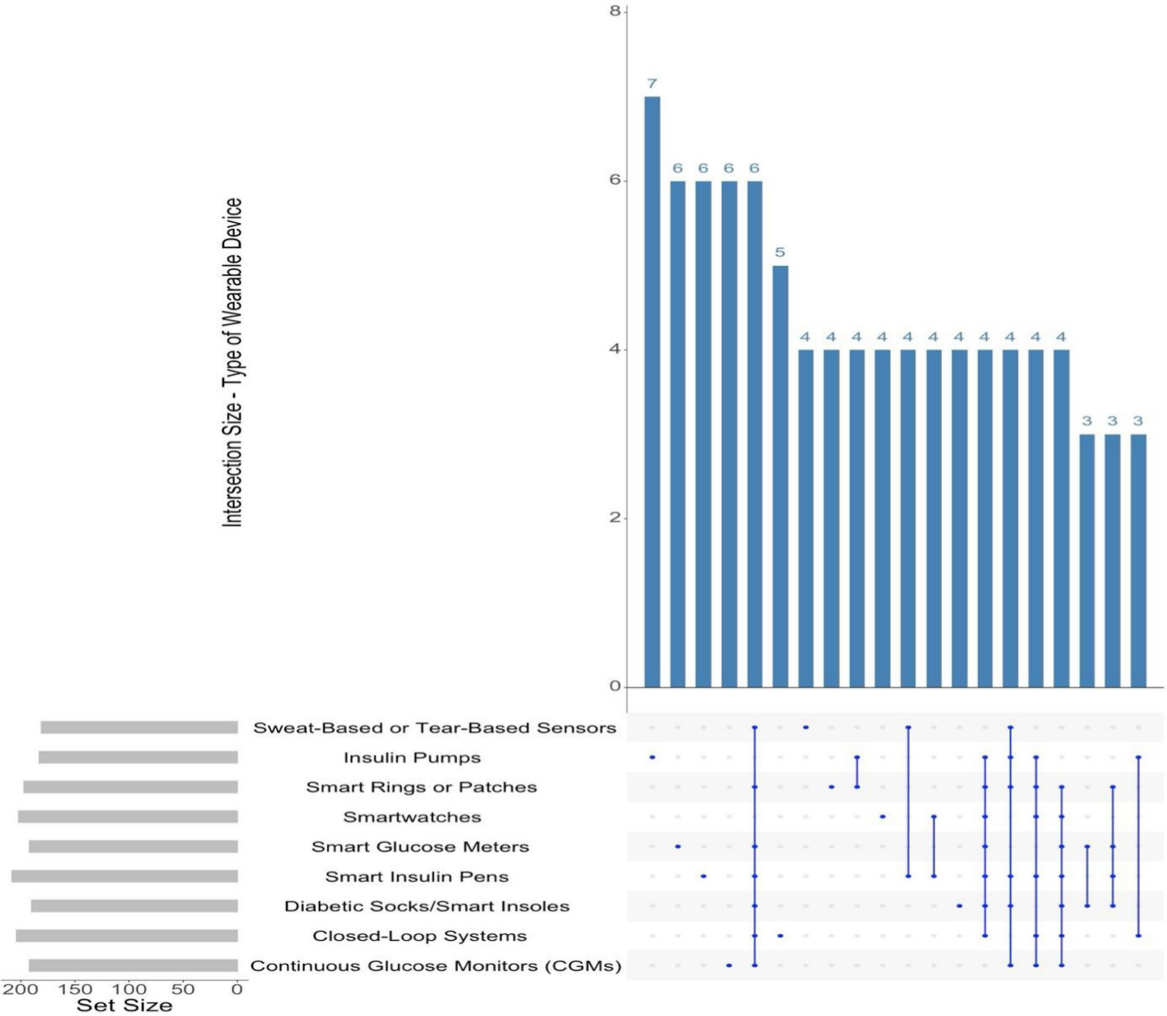
Types of wearable devices reported by participants.

The upset plot illustrates the responses of people with diabetes regarding their knowledge and recommendations of common wearable devices for diabetes management, based on a multiple-choice question. The data illustrates both individual and collective preferences for different types of devices (see Figure 3).

CGMs and smartwatches emerged as the most recognised and recommended devices, with endorsements from 180 and 170 participants, respectively. The results indicate the recognised application of CGMs and smartwatches in diabetes management, especially for real-time glucose monitoring and the tracking of health metrics.

The intersections indicate various combinations of device recommendations. One hundred twenty participants endorsed both CGMs and smartwatches, suggesting a preference for devices that synergistically assist in diabetes management. Additionally, 90 participants acknowledged a combination of continuous glucose monitors, smartwatches, and smart insulin pens, indicating a recognition of devices that integrate glucose monitoring, health tracking, and medication management. Nevertheless, a limited number of participants endorsed less conventional devices, including smart clothing or smart shoes, with fewer than 20 individuals supporting these options either individually or in combination with others.

Table 2 highlights participants’ responses to Likert-scale items offers insights into their perceptions of wearable devices that incorporate artificial intelligence (AI) for diabetes management. Participants indicated favourable views regarding the potential advantages of wearable devices, while also noting specific concerns.

**Table 2:** Participants’ perceptions of AI-integrated wearable devices for diabetes management (*N* = 418).

Question	Mean Score	Standard Deviation	Strongly Disagree (1)	Disagree (2)	Neutral (3)	Agree (4)	Strongly Agree (5)
I am aware that wearable devices can help monitor health metrics such as glucose levels and physical activity.	3.08	1.17	50 (12.22%)	77 (18.83%)	113 (27.63%)	129 (31.54%)	40 (9.78%)
I am familiar with the use of artificial intelligence (AI) in health-related applications and devices.	3.12	1.13	35 (8.56%)	88 (21.52%)	125 (30.56%)	114 (27.87%)	47 (11.49%)
I have seen or heard about wearable devices specifically designed for diabetes management.	3.07	1.17	46 (11.25%)	88 (21.52%)	108 (26.41%)	126 (30.81%)	41 (10.02%)
I believe AI tools in wearable devices can provide valuable recommendations for managing diabetes.	3.09	1.14	44 (10.76%)	80 (19.56%)	117 (28.61%)	132 (32.27%)	36 (8.80%)
I trust wearable devices that use AI to analyse my health data accurately.	3.09	1.15	46 (11.25%)	79 (19.32%)	117 (28.61%)	128 (31.30%)	39 (9.54%)
I believe wearable devices integrated with AI can help me manage my blood glucose levels more effectively.	4.2	0.97	8 (1.96%)	23 (5.62%)	43 (10.51%)	142 (34.72%)	193 (47.19%)
I feel that AI-based wearable devices can provide more personalized health insights compared to traditional methods.	4.16	1.03	15 (3.67%)	20 (4.89%)	39 (9.54%)	144 (35.21%)	191 (46.70%)
I believe wearable devices can help me track my physical activity, diet, and sleep patterns to support better diabetes management.	4.21	0.98	13 (3.18%)	17 (4.16%)	36 (8.80%)	150 (36.67%)	193 (47.19%)
Using AI in wearable devices can assist in early detection of health issues related to diabetes.	4.24	0.93	5 (1.22%)	25 (6.11%)	35 (8.56%)	146 (35.70%)	198 (48.41%)
I believe that wearable devices with AI can help reduce the frequency of clinic visits by providing remote health monitoring.	4.21	0.98	8 (1.96%)	25 (6.11%)	38 (9.29%)	141 (34.47%)	197 (48.17%)
I am concerned about the privacy of my personal health data when using wearable devices integrated with AI.	1.97	0.97	141 (34.47%)	185 (45.23%)	46 (11.25%)	27 (6.60%)	10 (2.44%)
I worry about the security of wearable devices and the possibility of data breaches.	1.99	1.03	160 (39.12%)	142 (34.72%)	67 (16.38%)	30 (7.33%)	10 (2.44%)
I feel that wearable devices may be too complex or difficult to use.	2.05	1.01	135 (33.01%)	172 (42.05%)	59 (14.43%)	32 (7.82%)	11 (2.69%)
I am concerned about the cost of wearable devices integrated with AI tools.	1.98	1.04	157 (38.39%)	158 (38.63%)	53 (12.96%)	27 (6.60%)	14 (3.42%)
I am worried that I may become too dependent on wearable devices for managing my diabetes.	2.07	1.06	141 (34.47%)	159 (38.88%)	63 (15.40%)	31 (7.58%)	15 (3.67%)
I would be willing to use a wearable device that integrates AI to support my diabetes management.	4.21	0.96	5 (1.22%)	28 (6.85%)	41 (10.02%)	139 (33.99%)	196 (47.92%)
I believe wearable devices would increase my confidence in managing my diabetes independently.	4.2	0.91	8 (1.96%)	13 (3.18%)	49 (11.98%)	158 (38.63%)	181 (44.25%)
If my healthcare provider recommended an AI-integrated wearable device, I would be more likely to use it.	4.21	0.94	6 (1.47%)	25 (6.11%)	37 (9.05%)	151 (36.92%)	190 (46.45%)
I believe wearable devices can enhance communication with my healthcare team by sharing real-time data.	4.22	0.93	6 (1.47%)	19 (4.65%)	47 (11.49%)	144 (35.21%)	193 (47.19%)
I am willing to try new technologies, such as wearable devices with AI, to improve my health outcomes.	4.14	0.98	8 (1.96%)	27 (6.60%)	41 (10.02%)	156 (38.14%)	177 (43.28%)

A significant majority of participants acknowledged the advantages of AI-integrated wearable devices. For instance, 47.19% of respondents strongly agreed, and 36.67% agreed that wearable devices assist in monitoring physical activity, diet, and sleep patterns to enhance diabetes management (mean = 4.21, SD = 0.98). In a similar vein, 48.41% of respondents strongly agreed and 35.70% agreed that wearable devices could aid in the early detection of diabetes-related health issues (mean = 4.24, SD = 0.93). A majority of participants expressed the belief that wearable devices would enhance their confidence in independently managing diabetes, with 44.25% strongly agreeing and 38.63% agreeing (mean = 4.20, SD = 0.91). Participants highlighted the importance of wearable devices in improving communication with healthcare providers through the sharing of real-time data, with 47.19% strongly agreeing and 35.21% agreeing (mean = 4.22, SD = 0.93).

Participants demonstrated a readiness to embrace wearable technologies, with 47.92% strongly agreeing and 33.99% agreeing to the use of AI-integrated wearable devices for diabetes management (mean = 4.21, SD = 0.96). Additionally, 46.45% of respondents strongly agreed, and 36.92% agreed that a recommendation from their healthcare provider would affect their likelihood of adopting these devices (mean = 4.21, SD = 0.94). This indicates the confidence participants have in expert advice when evaluating emerging technologies.

Participants, despite their favourable views, articulated significant concerns regarding wearable devices. Data privacy and security emerged as critical concerns, with 45.23% of respondents expressing disagreement and 34.47% indicating strong disagreement regarding their sense of security related to the privacy of personal health data when utilising these devices (mean = 1.97, SD = 0.97). In a similar vein, 34.72% of respondents strongly disagreed, while 39.12% disagreed concerning concerns about the potential for data breaches (mean = 1.99, SD = 1.03). Cost emerged as a significant concern, with 38.63% of respondents disagreeing and 38.39% strongly disagreeing regarding the affordability of wearable devices (mean = 1.98, SD = 1.04).

Participants offered varied responses to the open-ended question concerning the potential role of wearable devices in managing diabetes and daily medication adherence. The responses were classified into two primary themes: Reminder and Adherence Support, and Ease of Use and Feedback, with multiple subcodes identified within each theme (see Table 3).

**Table 3:** Thematic analysis of participants’ open-ended responses on the role of wearable devices in diabetes and medication management.

Themes	Codes	Sample Responses	Frequency
Reminder and Adherence Support	Remind me about medication/insulin doses	“It would be helpful to remind me about my insulin doses.” “Wearable devices can help me remember doses when I’m busy.”	15
	Track sugar levels easily	“I believe wearable devices can help me track my sugar levels more easily.” “I want to monitor trends over time easily.”	12
	Medication refill alerts	“If the device could alert me to refill my medication, that would be helpful.” “I often forget to refill on time.”	10
Ease of Use and Feedback	Simplify daily routine	“If it can sync with my phone and remind me, it would simplify my routine.” “I prefer fewer manual entries in apps.”	9
	Actionable feedback	“I want the device to provide actionable feedback, not just numbers.” “A summary of daily activity would help me plan better.”	8
	Integration with healthcare provider	“A device that integrates with my healthcare provider’s system would be valuable.” “Sharing data with my doctor easily is crucial.”	6

### Reminder and adherence support

Participants consistently emphasised the capacity of wearable devices to improve adherence to diabetes management protocols via reminders and tracking mechanisms. Medication or insulin dose reminders were the most frequently noted feature, referenced by 15 participants. Participants indicated that reminders for insulin doses would be beneficial, stating, “It would be helpful to remind me about my insulin doses” and “Wearable devices can help me remember doses when I’m busy.” This highlights the necessity for prompt reminders to maintain compliance with prescribed regimens.

Twelve participants also suggested the importance of easily tracking sugar levels. Participants indicated that wearable devices may enhance the monitoring of glucose trends over time, with one individual noting, “I believe wearable devices can help me track my sugar levels more easily.” Furthermore, 10 participants indicated a desire for medication refill alerts, underscoring the difficulty of recalling prescription refills. One participant stated, “An alert to refill my medication would be beneficial.” The findings highlight the significance of automated alerts and tracking functionalities in wearable devices for effective diabetes management.

### Meaningful use

Participants highlighted the significance of wearable devices in streamlining their routines and delivering actionable feedback. Nine participants recognised the capability of wearable devices to streamline daily routines by alleviating the manual demands of diabetes management. One participant stated, “If it can sync with my phone and provide reminders, it would simplify my routine.”

Moreover, actionable feedback was identified as a significant feature by eight participants. Respondents indicated a preference for meaningful insights over raw data, with one participant stating, “I want the device to provide actionable feedback, not just numbers.” Participants expressed appreciation for devices capable of generating summaries of daily activities to enhance their planning efforts.

Integration with healthcare providers was identified as a desirable feature by six participants. A participant noted, “A device that integrates with my healthcare provider’s system would be valuable.” This suggests a preference for wearable devices that allow for seamless data sharing with healthcare professionals, thereby enhancing personalised care and facilitating proactive management.

“In your opinion, how do you think wearable devices could help you in managing your diabetes and daily medication routines (including tracking, reminders, or refill assistance)?”

Participants articulated diverse concerns and challenges associated with the utilisation of wearable devices for diabetes and medication management. The responses were classified into two primary themes: Barriers and Concerns and Usability Issues, each emphasising particular challenges that participants experience or expect to face (see Table 4).

**Table 4:** Thematic analysis of participants’ perceived barriers and concerns regarding the use of AI-integrated wearable devices for diabetes management.

Theme	Code	Sample Responses	Frequency
Barriers and Concerns	Accuracy of data collection	“I’m worried about the accuracy of the data the device collects.” “Sometimes, I see discrepancies in readings.”	14
	Device malfunction during use	“I am concerned that the device might malfunction during a critical time.” “I’ve had devices stop working mid-day.”	11
	High cost of devices	“The cost might be too high for me to afford consistently.” “Subscriptions for app services add up quickly.”	10
	Privacy concerns	“Privacy is a concern. I do not want my health data to be accessible to unauthorized parties.” “What if my data is hacked?”	7
	Battery life issues	“The battery life of wearable devices can be an issue.” “I forget to charge it frequently, and it dies mid-day.”	9
Usability Issues	Uncomfortable to wear	“Wearing a device all day might be uncomfortable.” “It’s bulky and noticeable, making it inconvenient to wear outside.”	6
	Compatibility with mobile phones	“It may not work well with my phone if the software is not compatible.” “Some updates break compatibility with older phones.”	5
	Frequent calibration/updates	“I do not want to rely on a device if it requires frequent updates or calibration.” “Calibrating takes time and effort.”	4

### Barriers to use wearable devices

Accuracy of data collection was the primary concern, highlighted by 14 participants. Numerous participants raised concerns regarding the reliability of the data, with one participant noting, “I’m worried about the accuracy of the data the device collects,” while another pointed out inconsistencies in the readings. This suggests that participants emphasise the importance of trust in the reliability and accuracy of devices for effective diabetes management.

Eleven participants also reported concerns regarding device malfunction during use. One respondent expressed concern regarding potential device malfunction during critical periods. These concerns highlight the necessity for reliable and consistent device performance, particularly in the context of continuous glucose monitoring and medication reminders.

The high cost of devices was identified as a significant barrier by 10 participants. Respondents identified the affordability of devices and related subscriptions as significant challenges, with one remarking, “The cost might be too high for me to afford consistently.” Privacy concerns were expressed by seven participants, indicating worries regarding unauthorised access to personal health data. A participant enquired, “What are the implications if my data is compromised?” The responses indicate that affordability and data security are significant factors affecting participants’ willingness to adopt wearable devices.

Finally, nine participants identified battery life issues, expressing dissatisfaction with the limited duration of battery performance and the inconvenience associated with frequent recharging. One participant remarked, “I often forget to charge it, resulting in it dying during the day.” This indicates that enhanced battery longevity or reminders for device charging may enhance user satisfaction.

### Usability challenges

Participants expressed concerns regarding the usability of wearable devices, noting that 6 individuals found them potentially uncomfortable or bulky. A participant noted, “Wearing a device all day might be uncomfortable,” highlighting the significance of ergonomic design for everyday use.

Five participants noted that compatibility with mobile phones posed an additional challenge. Several respondents noted concerns regarding software updates and compatibility with older phone models, stating, “It may not work well with my phone if the software is not compatible.” This underscores the necessity for improved integration between devices and software across various user technologies.

Four participants expressed frustration regarding the necessity for frequent calibration or updates of wearable devices. A participant noted, “Calibrating takes time and effort,” suggesting that users favour devices necessitating minimal maintenance or adjustments.

“What concerns or challenges do you face, or do you anticipate facing, when using wearable devices for managing your diabetes and medications?”

## Discussion

This study examined people with diabetes’ perceptions, awareness, and attitudes regarding wearable devices and their function in managing chronic diseases, specifically diabetes. The findings indicated a notable awareness and favourable perception of wearable technologies among participants, with 74.2% recognising CGM as the most beneficial instrument for diabetes management. Devices including smartwatches, insulin pumps, and fitness trackers were commonly acknowledged, highlighting the adaptability of wearable technologies in meeting various health requirements. The findings are consistent with prior research demonstrating that wearable devices provide significant advantages in managing chronic diseases, including enabling real-time monitoring, enhancing adherence to treatment protocols, and promoting lifestyle changes (1, 3, 8).

The findings indicate multiple implications for integrating wearable devices into diabetes management. Participants indicated a clear preference for devices that offer real-time monitoring and actionable feedback, facilitating glucose level tracking and enabling timely interventions. These preferences align with previous research highlighting the importance of real-time data in enabling personalised care plans and early interventions for chronic disease management (1, 3). The study indicated that 77.8% of participants favoured devices suggested by healthcare professionals, highlighting the essential role of physicians in directing people with diabetes towards the adoption of these technologies. This finding corroborates the literature that emphasises increased physician engagement to improve patient confidence and trust in wearable devices (3, 5).

Participants emphasised that wearable devices enhance patient empowerment by fostering autonomy and engagement via personalised alerts and medication reminders. These observations align with findings from other studies indicating that wearables substantially improve patient engagement through the provision of real-time feedback and the encouragement of self-management behaviours (3, 8). Smartwatches connected to CGMs allow people with diabetes to monitor glucose levels discreetly, enhancing adherence to monitoring protocols and improving diabetes management.

Participants acknowledged multiple obstacles to the widespread adoption of wearable devices, despite their enthusiasm for them. Cost and affordability were identified by 64.5% of respondents as a major limitation, indicating concerns regarding the financial accessibility of these technologies. Previous studies have identified similar concerns, highlighting the cost of wearable devices as a significant barrier to patient adoption (10, 28). Furthermore, 71.3% of participants reported concerns regarding data privacy and security, aligning with existing literature that identifies privacy as a significant challenge in the adoption of digital health tools (3).

Usability issues, especially among older people with diabetes or individuals lacking familiarity with technology, have been identified as a persistent challenge. This finding is consistent with prior research highlighting the significance of user-friendly interfaces in promoting adoption, especially among people with diabetes with limited technological literacy (8). The identified barriers highlight the necessity of tackling socioeconomic and technological challenges to guarantee equitable access to wearable devices. Creating cost-effective alternatives and advancing educational programs to improve digital literacy may significantly contribute to the broader adoption and effectiveness of these technologies.

The findings align with previous studies highlighting the significant impact of wearable devices on managing chronic diseases. This study corroborates existing literature by highlighting the important function of real-time health monitoring in facilitating proactive care and diminishing the need for in-person consultations (1, 6). The concerns regarding cost and data privacy highlighted in this study reflect wider trends in patient apprehensions towards the adoption of digital health technologies. This study contributes to the literature by offering insights into the preferences and priorities of people with diabetes, specifically concerning the devices they consider most valuable. The identification of CGMs as the preferred device corresponds with their growing significance in diabetes care, while also highlighting aspects of patient awareness and acceptance.

The study’s findings indicate that 71.3% of participants reported satisfaction or high satisfaction with wearable devices for diabetes management, aligning with existing literature that underscores the favourable reception of such technologies among people with diabetes. Prior research indicates that wearable devices, including CGMs and smartwatches, improve people with diabetes’ capacity for real-time health monitoring, resulting in heightened engagement and improved adherence to treatment protocols. A study by (29) found that 78% of CGM users reported enhanced diabetes management and overall satisfaction, crediting their positive experiences to the convenience and real-time feedback offered by these devices.

The observed 20.6% neutrality and 8.1% dissatisfaction in the current study align with concerns identified in previous research. However, in a study by Megan and Georgina (30), many participants expressed apprehension regarding the adoption of wearable devices. Vigersky and Shrivastav (31) found that older adults and those less familiar with technology tended to remain neutral or dissatisfied, primarily due to difficulties in navigating complex device interfaces.

## Implications of study

This study provides important insights for healthcare professionals, policymakers, and technology developers aiming to enhance diabetes self-management through digital health tools. The findings highlight the growing openness among people with diabetes to adopt AI-integrated wearable technologies, particularly when these tools are perceived as trustworthy, accurate, and supportive of personalised care. The role of education in shaping perceptions further emphasises the need for tailored health literacy initiatives and inclusive design approaches that accommodate diverse user needs.

By focusing on people with diabetes in a Middle Eastern context a population often underrepresented in digital health research this study contributes to a more global understanding of wearable technology adoption. The contextual factors identified, including trust, perceived usefulness, and digital readiness, offer valuable guidance for refining existing adoption models and ensuring that future interventions are culturally relevant and patient-centred.

## Strength, limitations, and recommendations for future researches

This study offers a comprehensive exploration of the perceptions of people with diabetes towards AI-integrated wearable technologies, combining both quantitative and qualitative approaches. The mixed-methods design enhances the depth and validity of the findings, enabling not only statistical summarisation of attitudes but also rich contextual understanding through thematic analysis. The relatively large and diverse sample size improves the representativeness of the study and allows for meaningful interpretation across subgroups. Furthermore, the inclusion of underrepresented populations from a Middle Eastern context provides novel insights that contribute to a more global understanding of wearable technology adoption in diabetes management.

However, several limitations should be acknowledged. First, the study employed a convenience sampling strategy, which may introduce selection bias and limit the generalisability of the findings to the wider diabetic population. Participants may have been more motivated or technologically inclined, potentially skewing responses in favour of wearable adoption. Second, the sample was disproportionately composed of individuals with graduate-level education (70.33%), which may have influenced the level of digital health literacy and openness to emerging technologies. Third, the cross-sectional nature of the study captured perceptions at a single point in time, restricting the ability to assess changes in attitudes or behaviour over the long term. Additionally, while internal consistency was measured using Cronbach’s alpha, further statistical validation of the instrument, such as exploratory or confirmatory factor analysis, was not conducted. Inferential statistical techniques—such as chi-square tests or regression modelling—were also not employed, limiting the ability to determine associations between participant characteristics and their perceptions.

Finally, while the findings suggest a strong willingness among participants to adopt wearable technologies, these results should be interpreted with caution due to the self-selected nature of the sample and potential response bias associated with self-reported data. Future research should aim to validate these findings using probabilistic sampling methods and more representative demographic distributions. Longitudinal studies are also recommended to explore how perceptions and usage patterns evolve over time, particularly as AI-based tools become more integrated into routine diabetes care. Further, the inclusion of clinical outcome measures, alongside patient-reported perceptions, would help to triangulate the effectiveness and impact of wearable technologies in real-world settings.

## Conclusion

This study emphasises the increasing recognition and acceptance of wearable devices by people with diabetes, highlighting their potential to improve chronic disease management. It is crucial to address barriers including cost, data privacy, and usability to facilitate widespread adoption and ongoing engagement with these technologies. Future research should investigate the longitudinal outcomes associated with the use of wearable devices, evaluate their clinical efficacy, and determine strategies to improve accessibility and foster patient trust. Collaboration among healthcare providers, technology developers, and policymakers is essential for maximising the potential of wearable devices in transforming diabetes care.

## Data Availability

The raw data supporting the conclusions of this article will be made available by the authors, without undue reservation.
